# A survey on clinical presentation and nutritional status of infants with suspected cow' milk allergy

**DOI:** 10.1186/1471-2431-10-25

**Published:** 2010-04-23

**Authors:** Mário C Vieira, Mauro B Morais, José VN Spolidoro, Mauro S Toporovski, Ary L Cardoso, Gabriela TB Araujo, Victor Nudelman, Marcelo CM Fonseca

**Affiliations:** 1 Centre for Paediatric Gastroenterology, Hospital Pequeno Príncipe, R. Desembargador Motta, 1070, Curitiba-PR 80250-060, Brazil; 2 Department of Paediatrics, Universidade Federal de São Paulo, R. Botucatu, 598, São Paulo-SP 04023-062, Brazil; 3 Department of Paediatrics - School of Medicine, Pontifícia Universidade Católica, Av. Ipiranga, 6690, Porto Alegre-RS 90610-000, Brazil; 4 Department of Paediatrics - School of Medicine, Santa Casa de São Paulo, R. Cesário Motta Jr., 112 São Paulo-SP 01221-020 Brazil; 5 Department of Paediatrics, Instituto da Criança, Hospital das Clínicas, Faculdade de Medicina da Universidade de São Paulo, Av. Dr. Enéas Carvalho Aguiar, 647, São Paulo - SP 05403-000, Brazil; 6 Health Economics, Axia.bio Consulting, R. Setembrino Woitechumas, 38/4, São Paulo-SP 04563-090, Brazil; 7 Department of Paediatrics, Hospital Albert Einstein, Av. Albert Eisntein, 627, São Paulo - SP 05651-901, Brazil

## Abstract

**Background:**

Cow's milk is the most common food allergen in infants and the diagnosis of cow's milk allergy is difficult, even with the use of several diagnostic tests. Therefore, elimination diets and challenge tests are essential for the diagnosis and treatment of this disorder. The aim of this study is to report the clinical presentation and nutritional status of children evaluated by pediatric gastroenterologists for the assessment of symptoms suggestive of cow's milk allergy.

**Methods:**

An observational cross-sectional study was performed among 9,478 patients evaluated by 30 pediatric gastroenterologists for 40 days in 5 different geographical regions in Brazil. Clinical data were collected from patients with symptoms suggestive of cow's milk allergy. The nutritional status of infants (age ≤ 24 months) seen for the first time was evaluated according to z-scores for weight-for-age, weight-for-height, and height-for-age. Epi-Info (CDC-NCHS, 2000) software was used to calculate z-scores.

**Results:**

The prevalence of suspected cow's milk allergy in the study population was 5.4% (513/9,478), and the incidence was 2.2% (211/9,478). Among 159 infants seen at first evaluation, 15.1% presented with a low weight-for-age z score (< -2.0 standard deviation - SD), 8.7% with a low weight-for-height z score (< -2.0 SD), and 23.9% with a low height-for-age z score (< -2.0 SD).

**Conclusion:**

The high prevalence of nutritional deficits among infants with symptoms suggestive of cow's milk allergy indicates that effective elimination diets should be prescribed to control allergy symptoms and to prevent or treat malnutrition.

## Background

The incidence of food allergies has increased in several parts of the world, particularly in developed countries [1-6]. Cow's milk is the most common food allergen in infants and the diagnosis of cow's milk allergy is difficult, even with the use of several diagnostic tests. Therefore, elimination diets and challenge tests are essential for the diagnosis and treatment of this disorder [7,8]. Cow's milk and cow's milk-based infant formulas are the most frequently used substitutes for infants who are not breastfed. The growth and development of infants that are allergic to cow's milk may be compromised if an adequate substitute formula is not available [9-12]. The nutritional quality of cow's milk substitutes used for this group of patients has a crucial role in promoting appropriate growth and development [9-12].

Prior to 1950, the incidence of cow's milk allergy in the first year of life was low and affected about 0.1% to 0.3% of all infants. Prospective studies conducted in 1970 and 1988 showed that the incidence of cow's milk allergy reached 1.8% to 7.5%, a wide range explained by the differences in diagnostic criteria adopted in different studies [1,6]. Symptoms suggestive of cow's milk allergy are reported by parents of 5.0% to 15.0% of all infants [1]. These data draw attention to the importance of an accurate diagnosis of food allergy in order to avoid the use of elimination diets for prolonged periods of time in infants without a confirmed diagnosis.

The aim of this study was to describe the clinical presentation and nutritional status of children evaluated by pediatric gastroenterologists for the assessment of symptoms suggestive of cow's milk allergy.

## Methods

### Data collection

An observational cross-sectional study was performed applying a questionnaire to 30 pediatric gastroenterologists from 20 different cities in 11 states of five Brazilian geographical regions (North, Midwest, Northeast, Southeast, and South). Data were collected during 40 consecutive days in 2004. Information on the number of patients evaluated for food allergies, demographic data, clinical manifestations, time of onset of symptoms, management, and anthropometric data at birth and at the first consultation was obtained and recorded.

A total of 9,478 children were evaluated over the study period. Five hundred thirteen children were identified as having suspected food allergy. Each specialist evaluated a mean of 2.4 patients with cow's milk allergy per week, including 1.0 new case.

The nutritional status of infants (age ≤ 24 months) seen for the first time was evaluated according to z-scores for weight-for-age, weight-for-height, and height-for-age. Epi-Info (CDC-NCHS, 2000) software was used to calculate z-scores and the National Center for Health Statistics (NCHS) growth charts were used as reference values [13]. Considering that the nutritional needs and growth velocity are variable in the first 2 years of life, weight and length were presented according to 3 age groups: ≤ 6.0 months, 6.1 - 12.0 months, 12.1 - 24.0 months.

According to WHO, a nutritional deficit is defined when z-scores are below -2.0 standard deviations [14]. In a normal population distribution, 2.5% of the values are expected to be below this cut-off point; therefore, a rate > 2.5% suggests that the study population has a nutritional deficit in comparison with reference values.

SigmaStat ^® ^for Windows, version 3.1, was used for statistical analysis. Differences were classified as statistically significant when the p value was < 0.05. The study was approved by the Human Research Ethics Committee at the Hospital Pequeno Príncipe - Curitiba, Brazil.

## Results

Cow's milk allergy was suspected in 513 of 9,478 consultations in the pediatric age range. In 211 patients, suspicion of the diagnosis was made at the first medical visit, and in 302 patients the diagnosis was suspected at follow-up visits. Therefore, the prevalence of diagnosed and suspected cow's milk allergy in the study population was 5.4% (513/9,478), and the incidence was 2.2% (211/9,478). At first consultation, pediatric gastroenterologists agreed with the diagnostic hypothesis of cow's milk allergy made by the referring pediatrician in 82.0% of the cases. Among patients who had suspected cow's milk allergy (n = 211) at first consultation, 49.3% were referred to the pediatric gastroenterologist having already been switched to a substitute infant diet. The milk substitute most frequently prescribed by general pediatricians was a soy formula (58%). Other inappropriate treatments, including lactose-free cow's milk infant formula or goat' milk (11%), were also prescribed. Extensively hydrolyzed formulas were used in 11% of patients, as well as amino acid-based formulas (5%). A diet without milk substitutes was prescribed in 5% of patients.

The following results refer to new cases of infants (≤ 24 months of age) with symptoms suggestive of cow's milk allergy. Weight and length were recorded in 159 (90.8%) of the 175 patients < 24 months of age. One hundred thirty patients (81.7%) were evaluated in the 1^st ^year of life. In this group, 79 (49.7%) patients were evaluated in their 1^st ^six months of life and 51 (32.1%) in their 2^nd ^six months. Twenty-nine (18.2%) patients were evaluated in the 2^nd ^year of life.

Demographic data, weight and length at birth, clinical presentation, and duration of symptoms according to age group are presented in Table 1. The comparisons of gender and weight and height at birth in the three age groups did not show any statistically significant differences. The mean duration of symptoms showed a directcorrelation with the child's age.

**Table 1:** Gender, weight and length at birth, duration of symptoms, and clinical presentation according to age group

	Age group (months)			p
		
	≤ 6.0 (n = 79)	6.1 - 12.0 (n = 51)	12.1 - 24.0 (n = 29)	
**Gender (male/female)**^1^	39/40	30/21	16/13	0.561
**Birth weight (grams)**^2^	3015 (2821; 3348)	3020 (2793; 3364)	3122 (2955; 3370)	0.663
**Birth length (cm)**^2^	48.5 (47.3; 50.0)	49.0 (48.0; 50.0)	49.0 (47.1; 50.0)	0.901
**Duration of symptoms (months)**^2,3^	1.0 (1.0; 2.0)	5.0 (4.0; 7.0)	12.0 (10.0; 18.0)	<0.001
**Systemic symptoms**^1^	14 (17.7%)	18 (35.3%)	7 (24.1%)	0.075
**Digestive symptoms**^1,4^	76 (96.2%)	42 (82.4%)	23 (79.3%)	0.011
**Cutaneous symptoms**^1^	13 (16.5%)	10 (19.6%)	4 (13.8%)	0.789
**Respiratory symptoms**^1,4^	9 (11.4%)	16 (31.4%)	6 (20.7%)	0.019

1. Chi-square test

2. Median and 25th and 75th percentiles; Kruskal-Wallis test for multiple groups.

3. Dunn test for multiple comparisons for the variable "duration of symptoms"

- p < 0.05: (≤ 6.0 months) × (6.1 - 12.0 months);

- p < 0.05: (≤ 6.0 months) × (12.1 - 24.0 months);

- p < 0.05: 6.1 - 12.0 months) × (12.1 - 24.0 months);

4. Chi-square results:

Digestive symptoms: ≤ 6 months versus 6-12 months, p = 0.008; ≤ 6 months versus 12-24 months, p = 0.005; 6-12 months versus 12-24 months, p = 0.737.

Respiratory symptoms: ≤ 6 months versus 6-12 months, p = 0.005; ≤ 6 months versus 12-24 months, p = 0.304; 6-12 months versus 12-24 months, p = 0.216.

There was a predominance of gastrointestinal manifestations in all age groups (Table 1). Gastrointestinal symptoms were more frequent in the 1^st ^six months of life than in the other age groups (p < 0.05) whereas respiratory manifestations were more frequent in the 2^nd ^six months of life (p < 0.05).

The clinical manifestations of infants were grouped as follows and are presented in Table 2: systemic (weight loss, anorexia, and irritability); gastrointestinal; cutaneous (atopic dermatitis and urticaria); and respiratory. Exclusive gastrointestinal symptoms were observed in 74 patients (46.5%), exclusive respiratory symptoms in 7 patients (4.4%), exclusive cutaneous manifestations in 5 patients (3.1%), and systemic symptoms only in 2 patients (1.3%). Therefore, a total of 88 (55.3%) of the 159 patients had clinical manifestations in only one of the 4 categories. Gastrointestinal symptoms and 1 (n = 59) or 2 (n = 8) other clinical symptoms in another category (systemic, cutaneous, or respiratory) occurred in 67 patients (42.1%). Only 18 patients (11.3%) had no gastrointestinal manifestations.

**Table 2:** Clinical presentation of 159 infants (age ≤ 24 months) with symptoms suggestive of cow's milk allergy (each infant may have one or more clinical manifestation)

Clinical manifestation	N	%
**Gastrointestinal**	**141**	**88.7**
Vomiting and regurgitation	85	53.5
Colic	54	34.0
Diarrhea without blood	30	18.9
Constipation	25	15.7
Blood in stools with normal consistency	23	14.5
Bloody diarrhea	10	6.3
		
**Systemic**	**39**	**24.5**
		
**Cutaneous**	**29**	**18.2**
		
**Respiratory**	**31**	**19.5**

**Figure 1:**
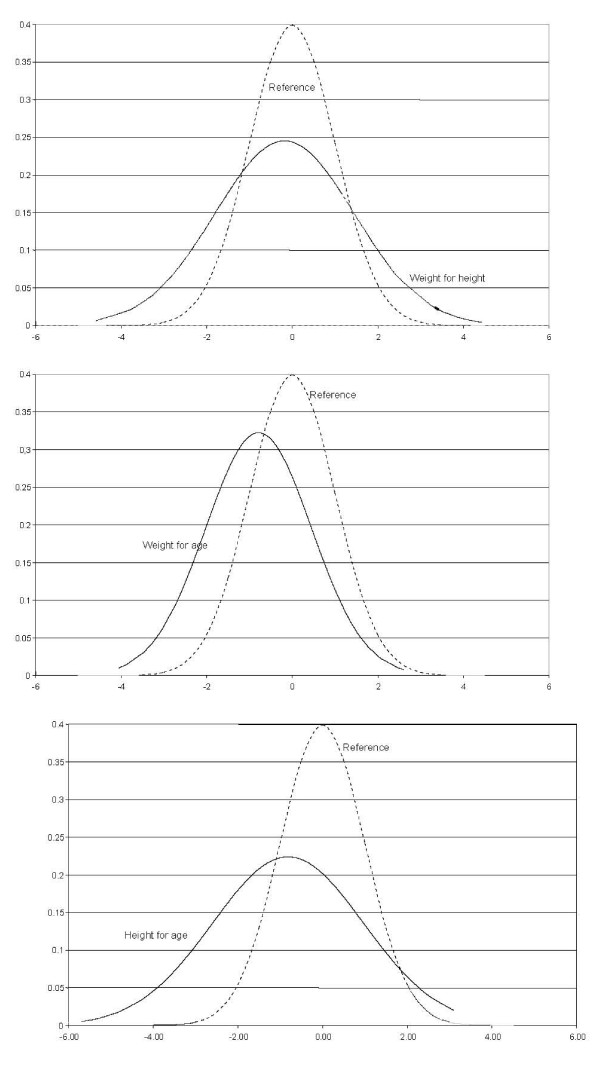
**Distribution of z-scores for weight-for-age, weight-for-height, and height-for-age according to normal reference values**.

The distribution of z-scores for weight-for-age, weight-for-height, and height-for-age of the 159 infants is presented in Figure 1. The analysis of z-score for weight-for-height revealed greater value dispersion, but the mean value was similar to the reference value. The weight-for-age curve followed an expected dispersion, but showed a deviation to the left. The height-for-age curve also showed a deviation to the left, but greater dispersion in comparison with the reference curve.

The distribution of patients according to anthropometric deficits confirmed by a z-score < -2.0 standard deviations is presented in Table 3. No statistically significant differences were found in the three age groups. The total number of infants with deficits involving weight-for-age, weight-for-height, and height-for-age were 15.1%, 11.3%, and 23.9%, respectively.

**Table 3:** Mean ± standard deviation (SD) of z-scores for weight-for-age, weight -for-height, and height-for-age, and number and percentage of infants with cow's milk allergy and nutritional deficits (z-score < -2.0 SD) according to age group

	≤ 6.0 m (n = 79)	6.1 - 12.0 m (n = 51)	12.1 - 24.0 m (n = 29)	p
**z-scores (mean ± SD)**^1^				
Weight-for-age	-0.70 ± 1.33	-0.83 ± 1.06	-0.9 ± 1.30	0.671
Weight-for-height	-0.21 ± 1.72	-0.08 ± 1.54	-0.27 ± 1.55	0.854
Height-for-age	-0.93 ± 1.82	-0.71 ± 1.78	-0.68 ± 1.76	0.724
				
**Deficit (z-score<-2.0 SD)**^2^				
Weight-for-age	13 (16.5%)	5 (9.8%)	6 (20.7%)	0.380
Weight-for-height	11 (13.9%)	4 (7.8%)	3 (13.8%)	0.556
Height-for-age	22 (27.8%)	12 (23.5%)	4 (13.8%)	0.315

1. One-way analysis of variance

2. Chi-square test

## Discussion

The incidence and prevalence of food allergies are believed to be increasing in several countries [1,2,15]. However, the comparison of data from different studies is difficult because criteria to diagnose food allergies, as well as the definitions of groups, vary substantially between studies. Therefore, rates vary from 35% when parental reports are used as criteria to define food allergies, to 1% when proper double-blind placebo-controlled food challenges (DBPCFC) are used [5,6].

A study was conducted in the Isle of Wight in the United Kingdom in order to establish the rates of objectively-assessed food allergies in the 1^st ^year of life and to compare this with the rate of parental reports. A cohort of 969 infants was recruited between September 2001 and August 2002. Symptoms of food allergies were reported by 132 parents (14.2%) at 3 months, 83 parents (9.1%) at 6 months, 49 parents (5.5%) at 9 months, and 65 parents (7.2%) at 12 months of age [4]. The cumulative incidence of reported parental perceived food hypersensitivities was 25.8% (250/969; 95% CI, 23.1% to 28.7%) by 12 months of age. Of these, only 14% and 6% were diagnosed with food allergies by means of open food challenges and DBPCFC, respectively. Thus, in this cohort, the incidence of food allergies by the age of 12 months was 2.6% (25/969; 95% CI, 1.7% to 3.8%) on the basis of open food challenge and 1.2% (12/969; 95% CI, 0.6% to 2.2%) on the basis of DBPCFC.

However, the actual occurrence of food allergies may be underestimated because challenge tests may be performed after the development of tolerance. This is a limitation for the method considered the gold standard for epidemiologic studies concerning food allergies. Therefore, the suggestion that the incidence of food allergies in the 1^st ^year of life ranges from 2% to 3% and symptoms compatible with food allergies found in 5% to 15% of infants may be reasonable and close to reality [1]. As distinct from other studies, we evaluated the prevalence and incidence of symptoms suggestive of cow's milk allergy according to data collected in the offices of pediatric gastroenterologists. The prevalence of suspected cow's milk allergy was 5.4% in all consultations, and the incidence of new cases was 2.2%. The pediatric gastroenterologists agreed with the diagnostic hypothesis made by the referring pediatrician in 82.0% of the consultations. Although the diagnosis of cow's milk allergy was not established by a milk challenge, patients were started on a cow's milk-free diet, which is the initial approach for this disorder. Nearly one-half of the patients had already received a prescription to eliminate cow's milk protein from their diets when they were seen for the first time by their pediatricians. However, many infants were being fed inappropriate substitutes, such as soy-based infant formulas, extracts with soybean proteins, goat's milk, or even lactose-free cow's milk formula. Only 10.0% of the infants received extensively hydrolyzed or amino acid-based formulas, considered adequate substitutes recommended for infants with cow's milk allergy according to the European Society for Pediatric Allergology and Clinical Immunology (ESPACI), the European Society for Pediatric Gastroenterology, Hepatology and Nutrition (ESPGHAN), and the American Academy of Pediatrics [7,16].

Approximately one-half of the infants in the study were younger than 6 months of age. In this age group, gastrointestinal manifestations occurred at a greater frequency than in the group of infants older than 6 months of age. As expected, digestive symptoms were the most common (88.7%), including regurgitation and vomiting, colic, diarrhea, and blood in stools. A variety of gastrointestinal allergic disorders typically affect in infants and children. Infants with allergic colitis present small amounts of blood mixed with mucus in their stools. Cow's milk-sensitive enteropathy may present with malabsorption leading to diarrhea and failure to thrive. The most serious form of gastrointestinal food allergy in infants is food protein-induced enterocolitis syndrome which has a symptom complex of profuse vomiting and diarrhoea, and potentially a sepsis-like clinical picture [17]

Cutaneous and respiratory symptoms were less frequent, possibly due to the fact that patients were referred to pediatric gastroenterologists. Although the predominant type of clinical manifestation may depend on the type of specialty care where the study patients are enrolled, there is a consensus that gastrointestinal or cutaneous symptoms are the predominant forms of presentation of cow's milk allergy.

A review of the literature did not yield studies with similar designs for comparisons. In a group of 204 infants with cow's milk allergy studied in the 1950s, the most common symptoms were atopic dermatitis in 43% of the cases, vomiting and regurgitation in 38%, colic in 31%, wheezing in 9%, irritability and anorexia in 22%, and constipation in 6% of the cases [18]. The most remarkable difference from our data is that we observed a greater frequency of allergic colitis and less atopic dermatitis. This current trend is supported by a study conducted in London reporting a reduction in cases of cow's milk allergy associated with intestinal malabsorption due to infectious gastroenteritis [19].

The mean z-score deviations, particularly for weight-for-age and height-for-age, suggest that failure to thrive or malnutrition may occur as a consequence of cow's milk allergy. The analysis of weight and height showed greater deficits (< -2.0 standard deviations) than expected (2.5%) according to the CDC-NCHS reference values (2000): specifically, 15.1% of weight-for-age, 11.3% of weight-for-height, and 23.9% of height-for-age z-scores (Table 3). The World Health Organization has recently issued new weight and height reference charts for infants who were exclusively breastfed [20]. These values were not used in our study because our patients were not breastfed, and this may help compare our data with other studies in the literature. A study reporting on a group of 26 Brazilian children demonstrated that 23% had a deficit in weight-for-age, 7.7% had a deficit in weight-for-height, and 11.5% had a deficit in height-for-age when the same diagnostic criteria and reference values were used. These patients were also shown to be receiving a diet with a lower energy intake and calcium content when compared with controls in the same age group and socioeconomic conditions. A nutritionally-inadequate elimination diet may lead to or aggravate anthropometric deficits of infants with symptoms suggestive of cow's milk allergy according to the few studies that investigated this issue [9-12]. Our results have shown that height-for-age deficit was the most predominant indicator of nutritional impairment in contrast with the expected predominance of weight-for-age deficit. Similar findings were reported in children with constipation secondary to cow's milk allergy, who presented a mean height-for-age deficit (-0.90 ± 1.24) more pronounced than the weight-for-age deficit (-0.67 ± 1.30) [21]. It may be possible that due to chronic inflammation secondary to milk allergy, linear growth may be impaired as it is observed in chronic liver disorders [22].

In this study, approximately half of the patients referred to the paediatric gastroenterologists were already switched to a substitute infant diet. However, only 16% of patients were receiving extensively hydrolyzed formulas or amino acid-based formulas. The duration of the substitute diet and the efficacy of treatment were variable. Therefore, the nutritional deficit observed in patients at the time of inclusion in the study may be attributable either to the use of inappropriate milk substitutes or to the insufficient duration of treatment for nutritional recovery.

Cow's milk allergy in infants is usually non-IgE mediated, and the diagnostic hypothesis should be raised using clinical symptoms and, if available, functional and morphological markers of gastrointestinal function. Since there are no effective laboratory methods for the diagnosis of this disorder, an elimination diet without allergenic proteins remains the first essential step to make a diagnosis of cow's milk allergy. Clinical follow-up to evaluate the response to treatment (elimination diet) is an essential step in the management of these patients [23]. After an initial phase of clinical and nutritional recovery, food challenges, when recommended, may provide a definitive diagnosis.

## Conclusions

Our data revealed the profile of infants with symptoms suggestive of cow's milk allergy and the presence of nutritional deficits in a considerable percentage of patients. These findings highlight the need to prescribe highly-effective elimination diets in order to control symptoms, to ensure fast nutritional recovery, and to avoid malnutrition. Further studies should be conducted to develop public healthcare strategies to provide adequate substitute diets and treat infants that have a diagnosis or symptoms suggestive of cow's milk allergy, a current concern in many countries [24].

## Competing interests

The authors have received fees from Support Advanced Medical Nutrition -Danone for technical assistance in this article.

## Authors' contributions

All authors read and approved the manuscript. **MCV: **study concept and design; analysis and interpretation of data; drafting of the manuscript; study supervision. **MBM: **study concept and design; analysis and interpretation of data; drafting of the manuscript; statistical analysis; study supervision. **JVNS: **study concept and design; analysis and interpretation of data; drafting of the manuscript; study supervision. **MST: **study concept and design; analysis and interpretation of data; drafting of the manuscript; study supervision. **ALC: **analysis and interpretation of data; drafting of the manuscript; critical revision of the manuscript for important intellectual content. **GTBA: **study concept and design; acquisition of data; analysis and interpretation of data; critical revision of the manuscript for important intellectual content; statistical analysis; study supervision. **VN: **analysis and interpretation of data; drafting of the manuscript; critical revision of the manuscript for important intellectual content. **MCMF: **study concept and design; acquisition of data; analysis and interpretation of data; critical revision of the manuscript for important intellectual content; statistical analysis; study supervision.

## Pre-publication history

The pre-publication history for this paper can be accessed here:

http://www.biomedcentral.com/1471-2431/10/25/prepub

## References

[B1] Host A Frequency of cow's milk allergy in childhood Ann Allergy Asthma Immunol 2002 89 6 Suppl 1 33 7 10.1016/S1081-1206(10)62120-5 12487202

[B2] Cataldo F Accomando S Fragapane ML Montaperto D SIGENP and GLNBI Working Groups on Food Intolerances Are food intolerances and allergies increasing in immigrant children coming from developing countries? Pediatr Allergy Immunol 2006 17 364 9 10.1111/j.1399-3038.2006.00421.x 16846455

[B3] Isolauri E Huurre A Salminen S Impivaara O The allergy epidemic extends beyond the past few decades Clin Exp Allergy 2004 34 1007 10 10.1111/j.1365-2222.2004.01999.x 15248842

[B4] Venter C Pereira B Grundy J Clayton CB Roberts G Higgins B Incidence of parentally reported and clinically diagnosed food hypersensitivity in the first year of life J Allergy Clin Immunol 2006 117 5 1118 24 10.1016/j.jaci.2005.12.1352 16675341

[B5] American College of Allergy, Asthma, & Immunology Food allergy: a practice parameter Ann Allergy Asthma Immunol 2006 96 3 Suppl 2 S1 68 16597066

[B6] Keil T Epidemiology of food allergy: what's new? A critical appraisal of recent population-based studies Curr Opin Allergy Clin Immunol 2007 7 259 63 10.1097/ACI.0b013e32814a5513 17489045

[B7] Host A Koletzko B Dreborg S Muraro A Whan U Agget P Dietary products used in infants for treatment and prevention of food allergy. Joint statement of the European Society for Pediatric Allergology and Clinical Immunology. Committee on hypoallergenic formulas and the European Society for Pediatric Gastroenterology Hepatology and Nutrition Arch Dis Child 1999 81 80 4 10.1136/adc.81.1.80 PMC1717972 10373144

[B8] Vandenplas Y Brueton M Dupont C Hill D Isolauri E Koletzko S Guidelines for the diagnosis and management of cow's milk protein allergy in infants Arch Dis Child 2007 92 902 8 10.1136/adc.2006.110999 17895338 PMC2083222

[B9] Tiainen JM Nuutinen OM Kalavainen MP Diet and nutritional status in children with cow's milk allergy Eur J Clin Nutr 1995 49 605 121 7588511

[B10] Arvola T Holmberg-Marttila D Benefits and risks of elimination diets Ann Med 1999 31 293 8 10.3109/07853899908995893 10480761

[B11] Medeiros LC Speridião PG Sdepanian VL Fagundes-Neto U Morais MB Nutrient intake and nutritional status of children following a diet free from cow's milk and cow's milk by-products J Pediatr (Rio J) 2004 80 363 70 15505731

[B12] Noimark L Cox HE Nutritional problems related to food allergy in childhood Pediatr Allergy Immunol 2008 19 188 95 10.1111/j.1399-3038.2007.00700.x 18257908

[B13] Centers for Disease Control and Prevention and National Center for Health Statistics 2000 CDC growth charts: United States 2008 http://www.cdc.gov/growthcharts

[B14] World Health Organization Physical status: the use and interpretation of anthropometry Technical Report Series, 854 1995 Geneve: WHO 8594834

[B15] The International Study of Asthma and Allergies in Childhood (ISAAC) Steering Committee Worldwide variation in prevalence of symptoms of asthma, allergic rhinoconjunctivitis, and atopic eczema: ISAAC Lancet 1998 351 9111 1225 32 10.1016/S0140-6736(97)07302-9 9643741

[B16] Committee on Nutrition. American Academy of Pediatrics Hypoallergenic infant formulas Pediatrics 2000 106 346 9 10.1542/peds.106.2.346 10920165

[B17] Sicherer SH Food allergy Lancet 2002 360 701 10 10.1016/S0140-6736(02)09831-8 12241890

[B18] Clein NW Cow's milk allergy in infants Pediatr Clin North Am 1954 4 949 62 10.1016/s0031-3955(16)30159-6 13204086

[B19] Walker-Smith J An eye witness perspective of the changing patterns of food allergy Eur J Gastroenterol Hepatol 2005 17 1313 6 10.1097/00042737-200512000-00008 16292083

[B20] de Onis M Garza C Onyango AW Borghi E Comparison of the WHO child growth standards and the CDC 2000 growth charts J Nutr 2007 137 144 8 17182816 10.1093/jn/137.1.144

[B21] Tahan S Motta MEFA Goshima S Daher S Naspitz CK Solé D Fagundes-Neto U Morais MB Chronic constipation secondary to cow's milk allergy affects nutritional status in children J Pediatr Gastroenterol Nutr 2004 39 S235

[B22] Sokol RJ Stall C Anthropometric evaluation of children with chronic liver disease Am J Clin Nutr 1990 52 203 8 2375285 10.1093/ajcn/52.2.203

[B23] Walker-Smith JA Diagnostic criteria for gastrointestinal food allergy in childhood Clin Exp Allergy 1995 25 Suppl 1 20 2 10.1111/j.1365-2222.1995.tb01128.x 8542455

[B24] Guest JF Valovirta E Modeling the resource implications and budget impact of new reimbursement guidelines for the management of cow milk allergy in Finland Curr Med Res Opin 2008 24 1167 77 10.1185/030079908X280455 18341760

